# Comparative transcriptome profiles of human dental pulp stem cells from maxillary and mandibular teeth

**DOI:** 10.1038/s41598-022-12867-1

**Published:** 2022-05-25

**Authors:** Thira Faruangsaeng, Sermporn Thaweesapphitak, Chompak Khamwachirapitak, Thantrira Porntaveetus, Vorasuk Shotelersuk

**Affiliations:** 1 International Graduate Program in Geriatric Dentistry and Special Patients Care, Faculty of Dentistry, Chulalongkorn University, Bangkok, Thailand; 2 Center of Excellence in Genomics and Precision Dentistry, Department of Physiology, Faculty of Dentistry, Chulalongkorn University, Bangkok, 10330 Thailand; 3 Center of Excellence for Medical Genomics, Medical Genomics Cluster, Department of Pediatrics, Faculty of Medicine, Chulalongkorn University, Bangkok, Thailand; 4 Excellence Center for Genomics and Precision Medicine, King Chulalongkorn Memorial Hospital, The Thai Red Cross Society, Bangkok, Thailand

**Keywords:** Transcriptomics, Reverse transcription polymerase chain reaction

## Abstract

The molecular control of tooth development is different between the maxilla and mandible, contributing to different tooth shapes and locations; however, whether this difference occurs in human permanent teeth is unknown. The aim of this study was to investigate and compare the transcriptome profiles of permanent maxillary and mandibular posterior teeth. Ten participants who had a pair of opposing premolars or molars extracted were recruited. The RNA obtained from cultured dental pulp stem cells underwent RNA-sequencing and qRT-PCR. The transcriptome profiles of two opposing premolar pairs and two molar pairs demonstrated that the upper premolars, lower premolars, upper molars, and lower molars expressed the same top-ranked genes, comprising *FN1, COL1A1, COL1A2, ACTB,* and *EEFIA1,* which are involved in extracellular matrix organization, immune system, signal transduction, hemostasis, and vesicle-mediated transport. Comparative transcriptome analyses of each/combined tooth pairs demonstrated that *PITX1* was the only gene with different expression levels between upper and lower posterior teeth. *PITX1* exhibited a 64-fold and 116-fold higher expression level in lower teeth compared with their upper premolars and molars, respectively. These differences were confirmed by qRT-PCR. Taken together, this study, for the first time, reveals that *PITX1* is expressed significantly higher in mandibular posterior teeth compared with maxillary posterior teeth. The difference is more evident in the molars compared with premolars and consistent with its expression pattern in mouse developing teeth. We demonstrate that differences in lower versus upper teeth gene expression during odontogenesis occur in permanent teeth and suggest that these differences should be considered in molecular studies of dental pulp stem cells. Our findings pave the way to develop a more precise treatment in regenerative dentistry such as gene-based therapies for dentin/pulp regeneration and regeneration of different tooth types.

## Introduction

Humans depend on teeth for eating, communication, and physical appearance. A missing tooth compromises human health physically and psychologically. Some organisms (e.g., sharks) can replace lost teeth throughout their lives^1,2^. However, human teeth cannot generate multiple teeth. Biologists and tissue engineers are working together to investigate the approach of regenerating dental pulp tissue and create new teeth in different tooth types and morphologies^3,4^.

Many studies have discovered that essential signaling pathways, such as the TGFβ, BMP, and Wnt pathways, are important for dentin repair and dentinogenesis; however, the complete process is not fully elucidated^5–7^. It is not well understood which proteins or genes regulate dentin repair, how dental pulp stem cells regenerate dentin tissue, or the different incisor, canine, premolar, or molar locations and morphologies^8,9^.

Given the anatomic and location differences between the maxillary and mandibular teeth, there may be differences in the gene expression patterns in human dental pulp stem cells (hDPSC) in the respective teeth. Pantalacci et al*.* has investigated and compared transcriptomes during the development of two morphologically distinct serial organs, the upper and lower first molars of the mouse^10^. The identity of different branchial arches is regulated by *Hox*, *Pbx*, and *Otx* genes. It is hypothesized that the expression of *Dlx* genes regulates intra-branchial arch identity. Depew et al*.* examined mice lacking *Dlx5* and *Dlx6* gene expression^11^. They concluded that loss of *Dlx5* and *Dlx6* resulted in a transformation of the lower jaw into an upper jaw and that the cellular identity within an arch relied on a nested pattern of *Dlx* expression. However, no comparative study of hDPSC has yet been made between maxillary and mandibular human teeth, especially those obtained within the same individuals. RNA expression levels and patterns are different between humans and animals and among different human subjects^12,13^. To limit the variation when using RNA sequencing to investigate gene expression, we evaluated teeth with the same tooth type from the maxilla and mandible from the same individual.

Tooth development is regulated by genetic and environmental factors. Genetics play a role in determining the shape, size, number, and position of the teeth^14,15^. In vivo and in vitro studies have found many genes that are important for tooth development; however, there is still a lack of knowledge the genes expressed in adult human teeth. The gene expression profile and complex regulatory mechanisms still require investigation.

RNA sequencing (RNA-Seq) is a method that provides insight into the transcriptome of cells^16,17^. Compared with microarray-based methods, RNA-Seq provides greater resolution and broad coverage of the transcriptome that allows the discovery of new genes involved in the biological process of interest. Transcriptomic techniques have been particularly useful in identifying the transcriptome responsible for phenotypes and gene function. The assembly of RNA-Seq reads is ideal for gene expression studies of non-existing or poorly developed genomic resources^18^.

This study evaluated and compared the gene expression profiles of the maxillary and mandibular human teeth to determine if there are differences in gene expression patterns in hDPSC. This information may be applicable to clinical problems, such as disturbed eruption, repairing a tooth defect, and tooth bioengineering.

## Results

### Isolated cells characteristics

The isolated hDPSC were spindle-shaped and had a morphology similar to fibroblasts. Using flow cytometry, the hDPSC were positive for mesenchymal stem cell markers, CD44, CD73, CD90, and CD105, but negative for the hematopoietic stem cell marker CD45 (Fig. S1).

### Highly expressed genes in the upper and lower posterior teeth

**Figure 1:**
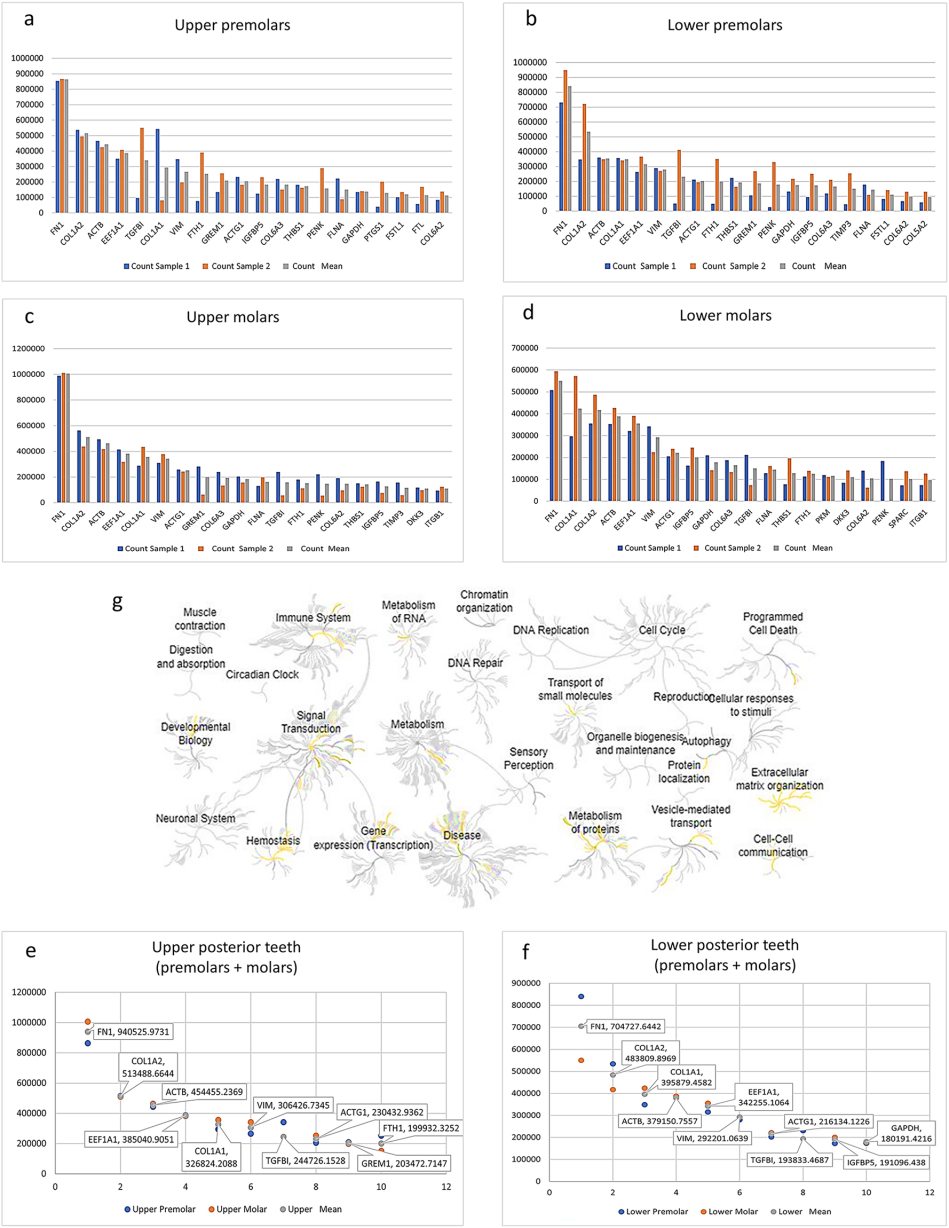
Top expression genes and related pathways. (**a**–**d**) Bar plots demonstrating the top 20 genes with the highest expression levels in the upper premolars (**a**), lower premolars (**b**), upper molars (**c**), and lower molars (**d**). (**e**) The reactome diagram presents the related pathways (in yellow) with the common 15 genes (*ACTB, COL1A1, COL1A2, COL6A2, COL6A3, EEF1A1, FLNA, FN1, FTH1, GAPDH, IGFBP5, PENK, TGFB1, THBS1,* and *VIM*) that were found in the upper premolars, lower premolars, upper molars, and lower molars. (**f**,**g**) Dot plots indicating the top ten most expressed genes in the upper posterior teeth (**e**) and lower posterior teeth (**f**).

The top 20 genes with the highest expression levels in the upper/lower premolars and molars are presented in Fig. 1a–d and Tables S1–S4. *FN1, COL1A1, COL1A2, ACTB,* and *EEFIA1* were the most highly expressed genes in all teeth. Among the top 20 genes, the upper premolars, lower premolars, upper molars, and lower molars expressed 15 genes in common, comprising *ACTB, COL1A1, COL1A2, COL6A2, COL6A3, EEF1A1, FLNA, FN1, FTH1, GAPDH, IGFBP5, PENK, TGFB1, THBS1,* and *VIM.* The reactome program analysis of these 15 genes showed that they are involved in signaling pathways such as extracellular matrix organization, immune system, signal transduction, hemostasis, and vesicle-mediated transport (Fig. 1g, Table S5).

When combining the premolars and molars as the posterior teeth, the ten most highly expressed genes in the upper posterior teeth were *FN1, COL1A2, ACTB, EEF1A1, COL1A1, VIM, TGFB1, ACTG1, GREM1,* and *FTH1* (Fig. 1e), and those in the lower posterior teeth were *FN1, COL1A2, COL1A1, ACTB, EEF1A1, VIM, ACTG1, TFGB1, IGFBP5,* and *GAPDH* (Fig. 1f). *FN1, COL1A2, ACTB, EEF1A1, COL1A1, VIM, TFGB1,* and *ACTG1* were observed in both the upper and lower posterior teeth.

### Differential gene expression between the upper and lower posterior teeth (premolars + molars)

**Figure 2:**
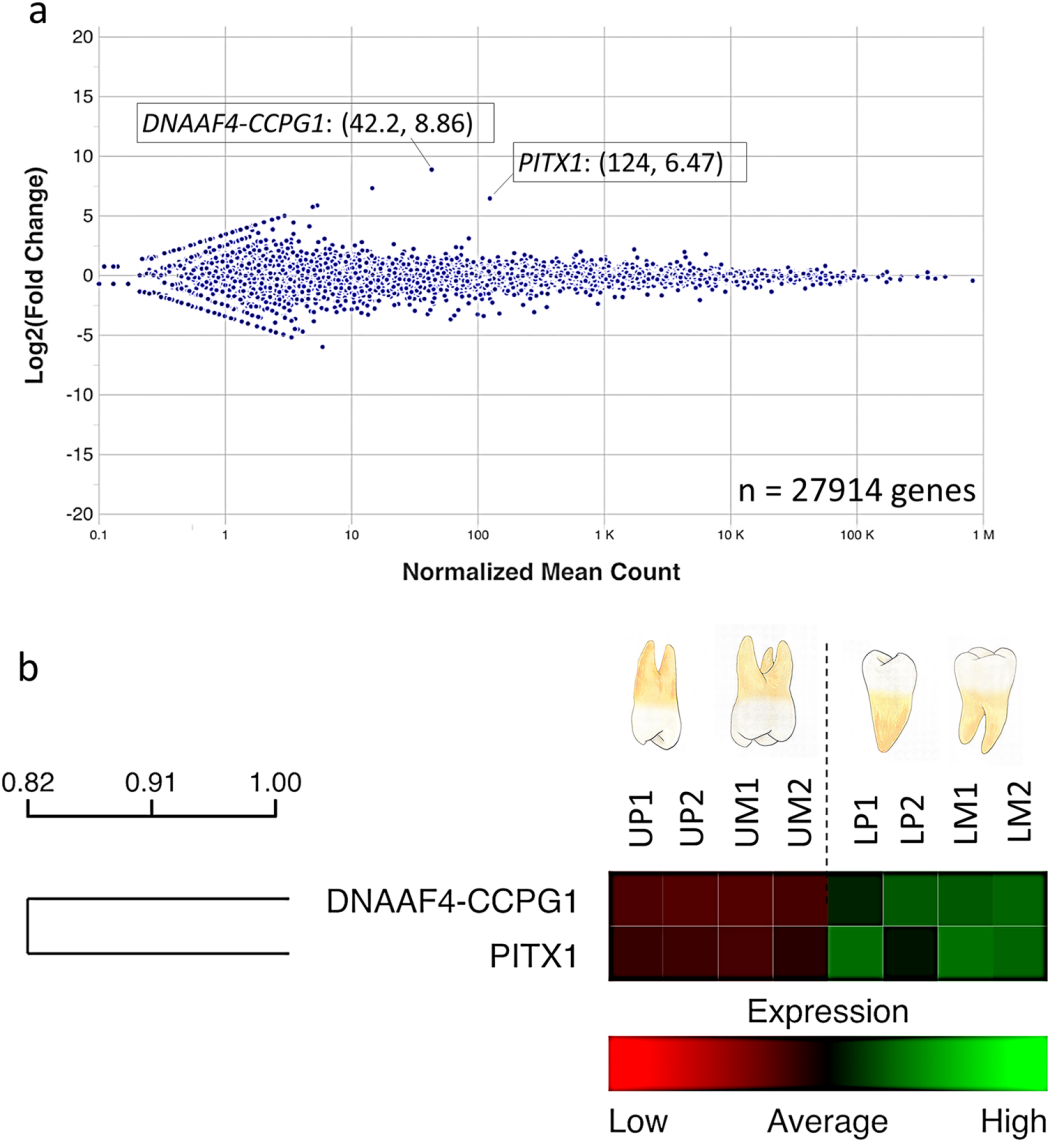
Differential gene expression between upper and lower posterior teeth (2 pairs of premolars + 2 pairs of molars). (**a**) MA-plot indicating the overall differential genes between the upper and lower posterior teeth. (**b**) Hierarchical cluster heat maps of differential expressed genes in 4 pairs of teeth reveal that two genes, *PITX1* and *DNAAF4-CCPG1*, had true significance and log2FC of ≤ − 2 or ≥ 2. One row in the heatmap represents one gene. The intensity of the color indicates the expression level, with green representing high expression level and red representing low expression level.

The data of 4 pairs of teeth comprising the premolar pair 1 (P1), premolar pair 2 (P2), molar pair 1 (M1), and molar pair 2 (M2), were combined and analyzed. The upper teeth of each pair (UP1, UP2, UM1 and UM2) were used as a control group, whereas the lower teeth (LP1, LP2, LM1, LM2) served as a comparison group. The total gene count was 27,914 genes (Fig. 2a). After excluding the genes with low counts, 19,372 genes were used for further analysis. The Heat map (Fig. 2b) demonstrates genes with true significance (*p* value ≤ 0.05 and FDR ≤ 0.05) and log2FC ≤ − 2 or ≥ 2. Only the *PITX1* and *DNAAF4-CCPG1* genes were significantly upregulated in the lower posterior teeth compared with the upper posterior teeth (Table S6).

### Differential gene expression between the upper and lower premolars

Considering each pair of premolars, P1 had 62 genes and P2 had 172 genes with true significance and log2FC ≤ − 2 or ≥ 2 (Table S7).

**Figure 3:**
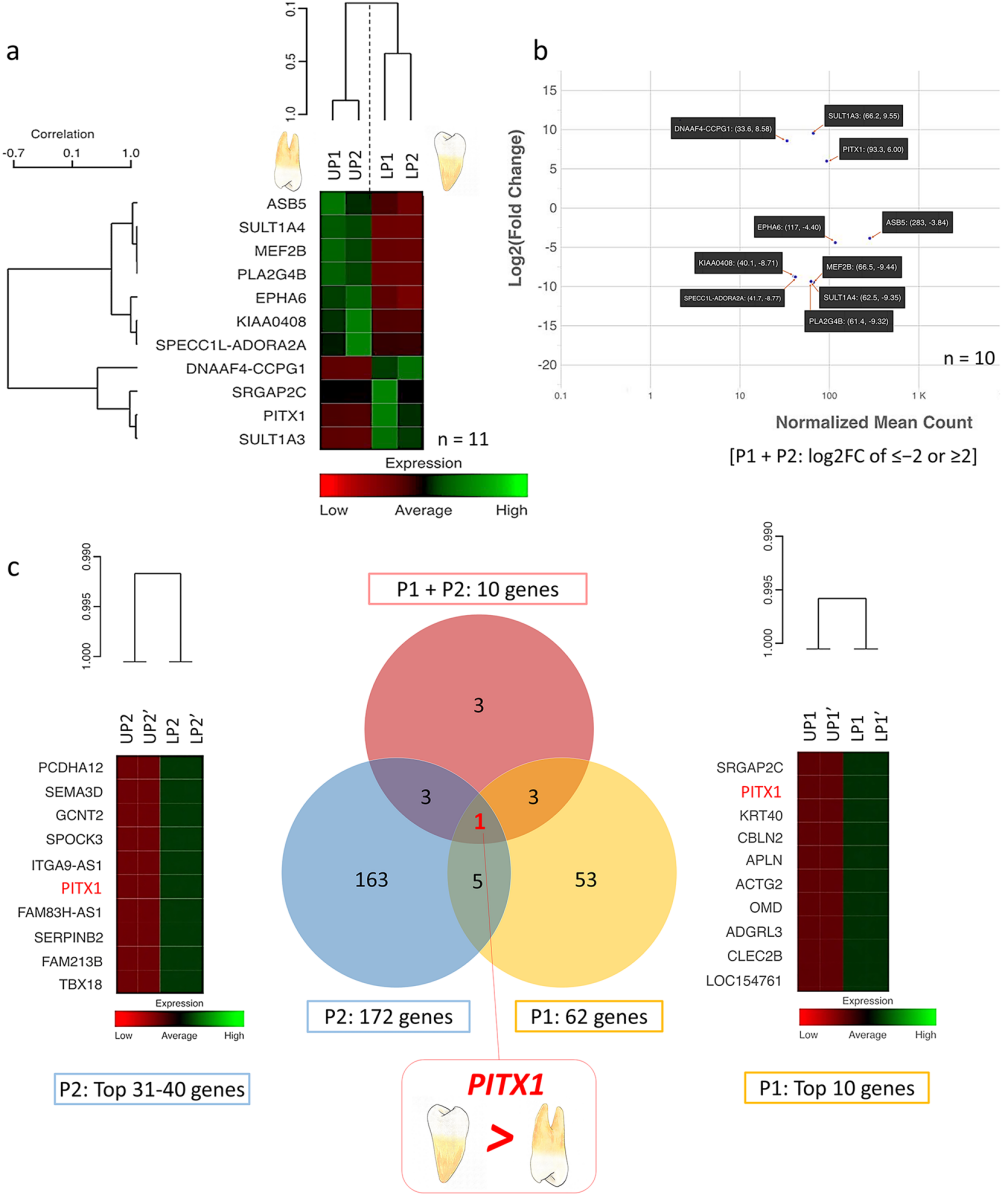
Differential gene expression between upper and lower premolars. (**a**) Heatmap presenting 11 genes having true significance and differential expression between upper and lower premolars in P1 + P2. (**b**) MA-plot showing 10 differential genes with log2FC of ≤ − 2 or ≥ 2. (**c**) Venn diagram of differential expression genes between upper and lower premolar teeth showing that only *PITX1* had significantly higher expression in the lower premolars compared with the upper premolars in all sets of premolar pairs.

To validate the analysis result of each premolar pair, the P1 and P2 data were combined and analyzed. After excluding genes with low counts, 11 genes had true significance shown in the heat map (Fig. 3a) and 10 genes had log2FC of ≤ − 2 or ≥ 2, comprising *SULT1A3, DNAAF4-CCPG1, PITX1, ASB5, EPHA6, KIAA0408, SPECC1L-ADORA2A, PLA2G4B, SULT1A4, MEF2B* (Fig. 3b, Tables S7, S8).

 When comparing P1, P2 and P1 + P2, only *PITX1* was found to be significantly different between the upper and lower premolars in all sets of analyses. *PITX1* had the 2nd highest expression level in P1 and the 36th in P2 (Fig. 3c). Detailed analysis of *PITX1* indicated that *PITX1* had a significantly higher expression, 2^6^ or 64-fold change, in the lower premolars compared with that in the upper premolars (Table S9). The *DNAAF4-CCPG1* gene that had a significant difference in the 4 teeth pairs combined analysis was also evaluated for its expression in premolar pairs. *DNAAF4*-*CCPG1* demonstrated significant differences in P2 and P1 + P2, but not in P1 (Table S10).

### Differential gene expression between the upper and lower molars

**Figure 4:**
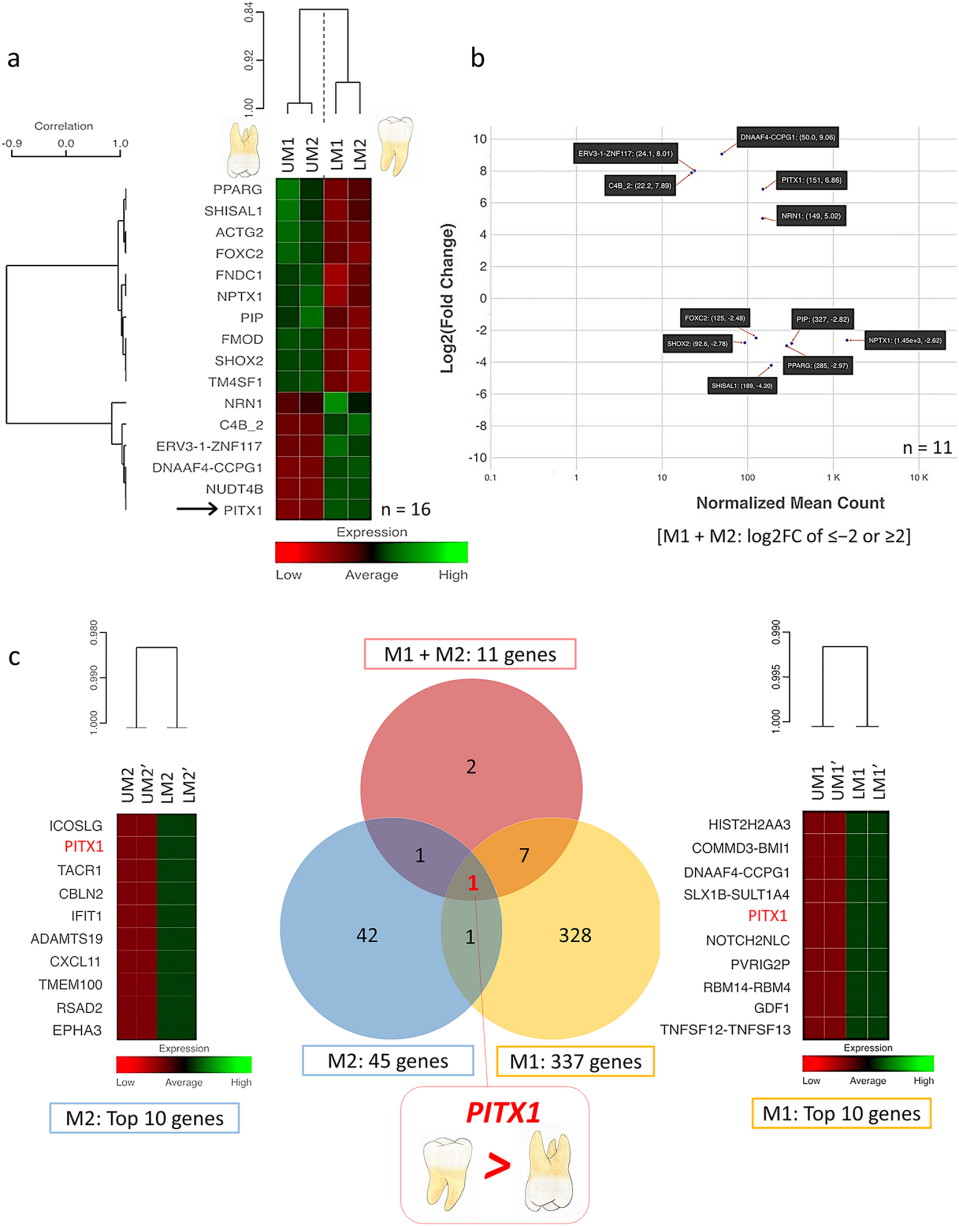
Differential gene expression between upper and lower molars. (**a**) Heatmap showing the 16 genes having true significance and differential expression between upper and lower premolars in P1 + P2. (**b**) MA-plot demonstrating 11 differential genes with log2FC of ≤ − 2 or ≥ 2. (**c**) Venn diagram of differential expression genes between upper and lower molar teeth shows that only *PITX1* had significantly higher expression in the lower molars compared with the upper premolars in all sets of molar pairs.

14,771 genes (Molar pair 1, M1) and 11,398 genes (Molar pair 2, M2) were analyzed after the genes with low counts were excluded. M1 had 337 genes and M2 had 45 genes with true significance and log2FC of ≤ − 2 or ≥ 2 (Table S11). Next, the M1 and M2 data were combined and analyzed, 16 genes had true significance shown in the heat map (Fig. 4a) and 11 genes had log2FC of ≤ − 2 or ≥ 2, comprising *DNAAF4-CCPG1, ERV3-1, ZNF117, C4B_2, PITX1, NRN1, FOXC2, NPTX1, SHOX2, PIP, PPARG,* and *SHISAL1* (Fig. 4a,b and Table S12). Comparing M1, M2, and M1 + M2, only *PITX1* was differentially expressed between the upper and lower molars in all sets of analyses. *PITX1* had the 5th highest expression level in M1 and the 2nd in M2 (Fig. 4c). The *PITX1* gene demonstrated a significantly higher expression, exhibiting a 2^6.86^ or 116-fold change, in the lower molars compared with that in the upper molars (Table S13) while *DNAAF4-CCPG1* presented significant differences in M1 and M1 + M2, but not in M2 (Table S14). Of note, the rank of *PITX1* expression in each tooth sample are shown in Table S15.

### Validation of RNA-Seq by quantitative real-time PCR

**Figure 5:**
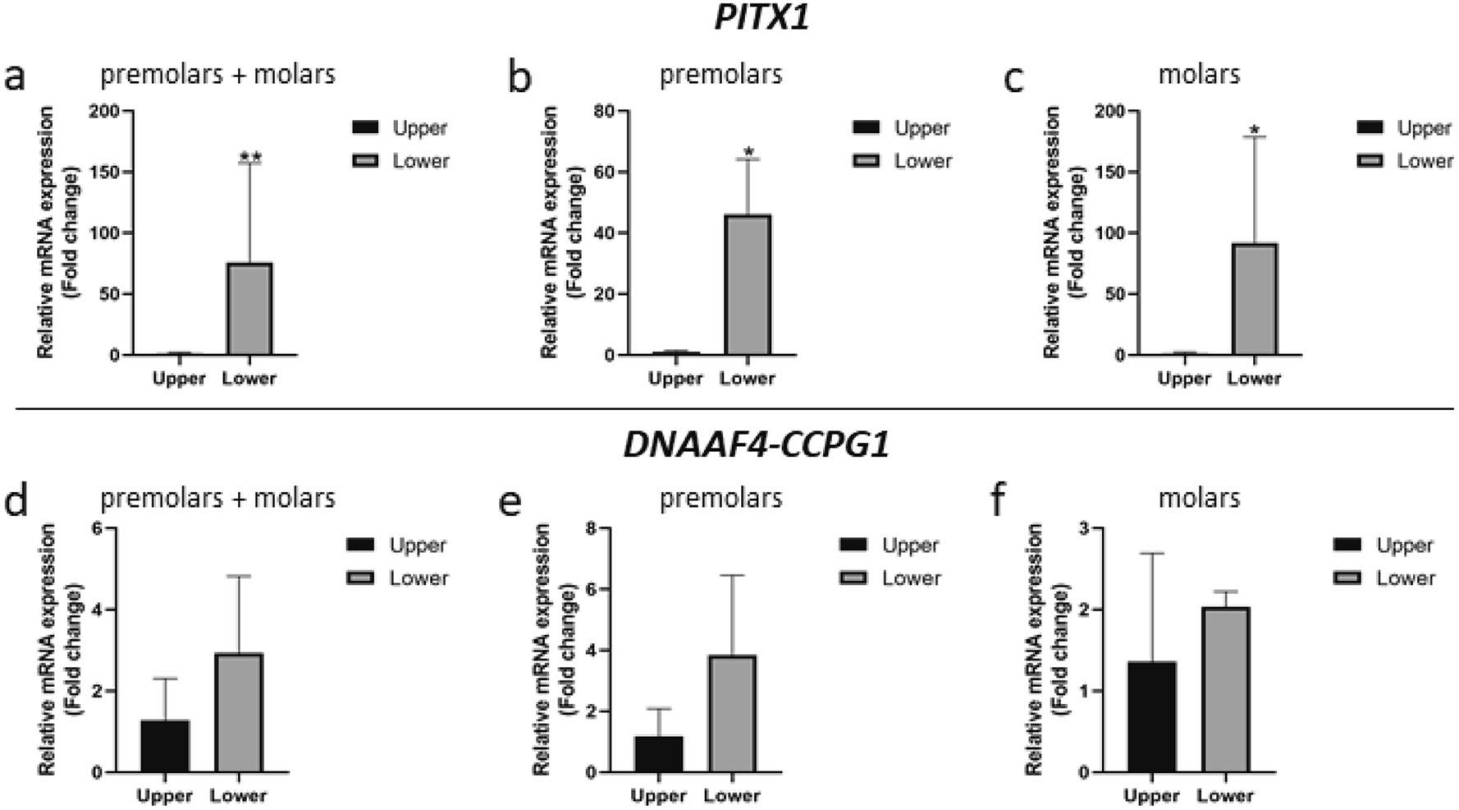
Quantitative real-time polymerase chain reaction. (**a–c**) qRT-PCR shows that *PITX1* was significantly higher expressed in the lower teeth compared with the upper teeth. (**d**,**e**) The expression of *DNAAF4-CCPG1* was not significantly different between the lower and the upper posterior teeth.

To validate the RNA-Sequencing data, the genes that had true significance at log2FC ≤ − 2 or ≥ 2 in all types of tooth pairs analyses (P1 + P2, M1 + M2, and P1 + P2 + M1 + M2) were selected for qRT-PCR. The expression levels of *DNAAF4*-*CCPG1* and *PITX1* were examined. The RNA samples for qRT-PCR were obtained from the other three pairs of premolars and three pairs of molars. *PITX1* had a significantly higher expression, a 46-fold-change, in the lower premolars than that in the upper premolars and a 92-fold change in the lower molars compared with the upper molars (Fig. 5a–c, Table S16, S17), consistent with the RNA-Seq results. In contrast, *DNAAF4-CCPG1* did not demonstrate significant differences in any tooth pairs analyses (Fig. 5d–f).

## Discussion

This study investigated the differential gene expression profiles between the upper and lower teeth using RNA-Sequencing in the hDPSC from two pairs of premolars and two pairs of molars, validated by qRT-PCR in the cells from the other three pairs of premolars and three pairs of molars. RNA-Seq, a high-throughput sequencing method, provides insight into the transcriptome of a cell and is one of the most common applications to study differential gene expression^19^. To limit the variation when using RNA-Seq to investigate gene expression, teeth with the same tooth type from the maxilla and mandible from the same individual were used. Our results indicated that in the hDPSC of the posterior teeth, only *PITX1* had a significantly higher expression in the lower teeth compared with the upper teeth in all 4 teeth pairs when analyzed separately. In contrast, *DNAAF4-CCPG1* did not have true significance (*p* value ≤ 0.05 and FDR ≤ 0.05) in some pairs of teeth and did not show significant differences by qRT-PCR. The significant difference in *DNAAF4-CCPG1* expression was discovered when all 4 pairs were analyzed together.

**Figure 6:**
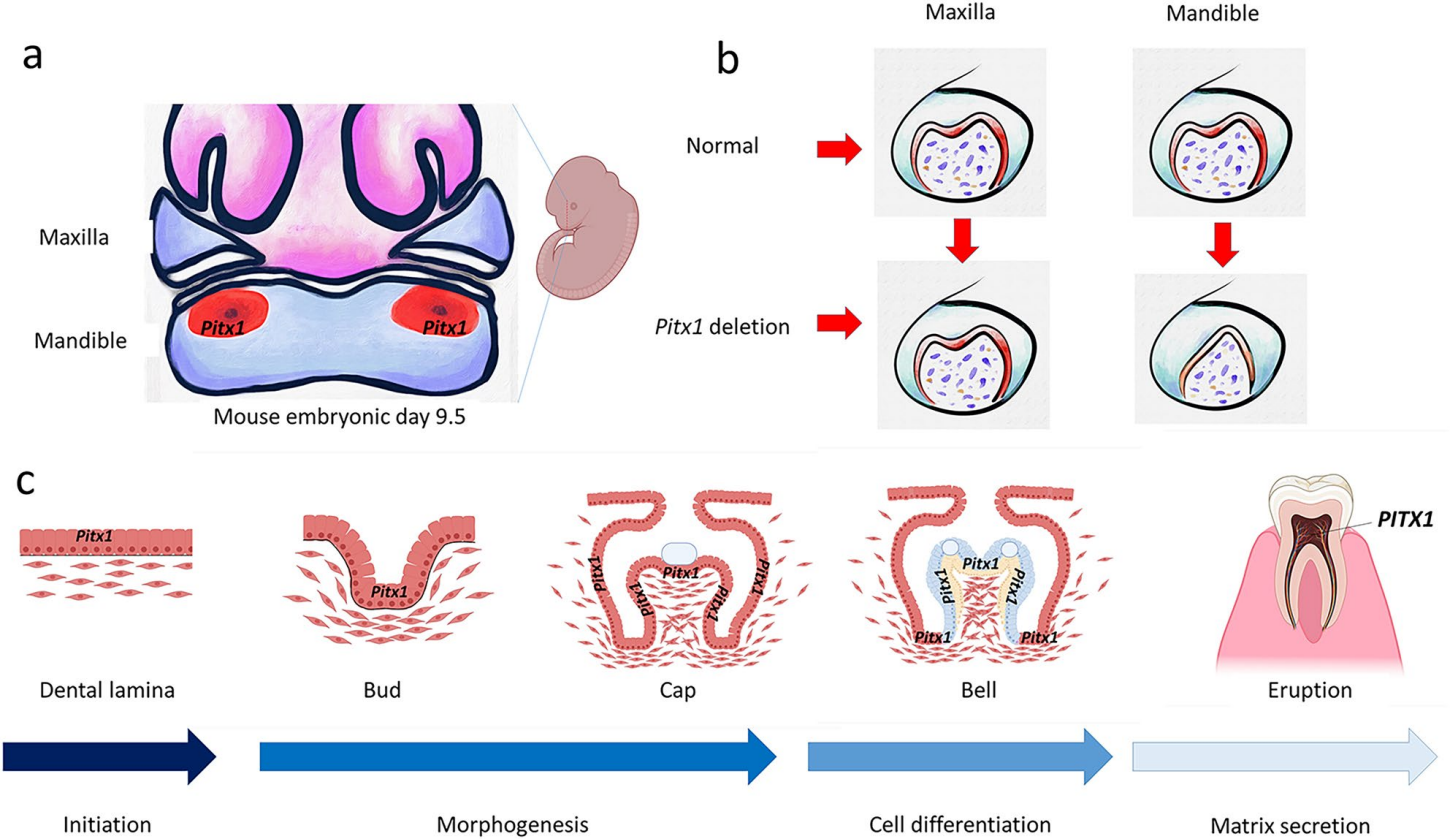
*PITX1* expression in the teeth. (**a**) *Pitx1* in mouse embryo is expressed in the mandibular mesenchyme where molars develop. (**b**) A picture demonstrates normal morphology of developmental maxillary and mandibular first molar of mouse embryo and the abnormal tooth development of the mandibular first molar (a presence of one cusp) in mouse embryo with *Pitx1* gene deletion. (**c**) Schematic diagrams showing the stages of normal tooth development and *PITX1* expression.

*PITX1* (Paired Like Homeodomain 1) is a gene that codes for a protein controlling pituitary development and hindlimb tissue patterning. *PITX1* is located on mouse chromosome 13 (11,147 bp long) and on human chromosome 5 (6495 bp long)^20^. During embryonic and postnatal development in mice, *Pitx1* is minimally expressed in the distal part of the mandible where the incisors develop; however, it is strongly expressed in the proximal part where the molars develop. *Pitx1* is enriched in mouse developing lower molars^21^. Micrognathia and mandibular molar deformation are the result of *Pitx1* deletion. *Pitx1* expression persists in the dental epithelium at all stages of odontogenesis and it interacts with *Tbx1* and *Barx1*. *Pitx1* mutant mice had normal maxillary molar development, but abnormal morphology of the mandibular molars, indicating that *Pitx1* plays an important role in mandibular molar development (Fig. 6)^22,23^.

Consistent with a previous study in murine developing teeth, our study found that the *PITX1* gene was expressed in the permanent posterior teeth and demonstrated higher expression in the lower teeth than that in the upper teeth (Fig. 6). We found that the differential expression of *PITX1* in the lower premolar teeth was ~ 64-fold higher by RNA-Seq and 46-fold higher by qRT-PCR compared with that in the upper premolar teeth (Tables S7, S13). *PITX1* in the lower molar teeth was ~ 116-fold higher by RNA-Seq and 92-fold higher by qRT-PCR compared with that in the upper molar teeth (Tables S11, S14). Our results indicate that the more posterior the opposing teeth are, the difference in the *PITX1* level between the upper and lower teeth is more evident.

Our results demonstrated that the most highly expressed genes in the posterior teeth were *FN1, COL1A1, COL1A2, ACTB*, and *EEF1A1. FN1, COL1A1*, and* COL1A2*, which are essential genes during tooth development, could still play role in permanent teeth. Fibronectin (*FN1*) is highly expressed in odontoblasts and is involved in extracellular matrix organization^24^. *FN1* also participates in cell movement via actin organization in the primary tooth buds^25^. Furthermore, *FN1* is a biomarker for head and neck squamous cell carcinoma due to its strong association with epithelial–mesenchymal transition and tumor invasion/metastasis^26^.

Fibronectin has been identified in the dental pulp and predentin at various phases of dentinogenesis. It is also detected in the basement membrane, which separates the inner enamel epithelium from the underlying dental mesenchyme, and in mantle predentin throughout tooth development^27,28^. Fibronectin is involved in the early phases of osteogenesis and collagen fibrillogenesis that is important in mineral nucleation^29^.

Collagen type 1, alpha 1 and collagen type 1, alpha 2 (*COL1A1* and *COL1A2*) have important roles in osteoblast and odontoblast activity^30,31^. *COL1A1* and *COL1A2* encode for type 1 collagen, which is the most common collagen type in humans^32^. Variations in *COL1A1* and *COL1A2* are associated with osteogenesis imperfecta and dentinogenesis imperfecta causing brittle dentin^33–36^. *Col1a1* mutant mice have a mandibular side shift and short craniofacial, maxillary, and mandibular morphometric indices, as well as a class III dental occlusion. Moreover, the mutants develop a larger periodontal space with altered vascularization and anomalies in dentin structure and mineralization^37^.

Beta-actin is encoded by the *ACTB* gene. Found in almost every cell type, actin is the most abundant protein and essential for cell structure, development, morphogenesis, cell migration, and cellular homeostasis. Beta-actin is a major cytoskeletal filament protein and an important player in cell motility and migration, and gene expression^38,39^.

The eukaryotic translation elongation factor 1 alpha 1 (*EEF1A1*) encodes an isoform of the alpha subunit of the elongation factor-1 protein, which is the second most abundant protein (1–3% of total protein) after actin. EEF1A1 is a GTP-binding protein, responsible for the delivery of aminoacylated-tRNA to the ribosome for decoding mRNA during protein synthesis and is involved in nervous system development^40,41^. Overall, the *FN1, COL1A1, COL1A2, ACTB*, and *EEF1A1* genes that are highly expressed in the posterior teeth are involved in extracellular matrix organization, signal transduction, vesicle-mediated transport, immune systems, and hemostasis that are essential for normal function of the dental pulp.

The strength of this study is that the upper and lower teeth were obtained from the same individual simultaneously. This limits the inter-subject variability of gene expression. Future studies should investigate other pairs of upper and lower teeth, such as incisors and canines in the same individual. Our findings demonstrate that *PITX1* is predominantly expressed in the lower posterior teeth compared with the upper posterior teeth. However, the incisors and canines may have different gene expression patterns between upper and lower teeth.

This is the first study in human permanent teeth to reveal differential gene expression between the upper and lower teeth in the same individual. The present results may promote further investigation into the gene expression between the upper and lower teeth in all tooth types (incisors, canines, premolars, and molars) and the function/molecular roles of genes and regulatory mechanisms in hDPSC.

This research provides new gene expression data in hDPSC isolated from permanent teeth. These gene expression data can be used for comparison with that in developing teeth and in patients with congenital tooth defects, expanding the understanding of the molecular physiology of hDPSC. Regenerative dentistry relies on the understanding of the biological mechanisms of tissue healing and repair. Regeneration of the dentin–pulp complex involves the migration, proliferation, and differentiation of hDPSC in dentin formation and inflammatory response. The knowledge about gene profiling and related signaling pathways in permanent teeth paves the way to explore new dental treatment approaches by targeting the interactions between tooth tissue and bioactive molecules. These findings may advance the knowledge about genetic engineering and can be used to comprehend the pulp cells’ biological behavior to ensure success when applied in stem cell therapy. Moreover, each tooth has its own shape and size. This is possibly due to each tooth has different set of genes essential for its own growth and development. In the future, when we realize all of these genes, we might be able to in vitro generate a certain tooth specific for an individual who needs one.

## Conclusion

Using RNA-Seq to study the gene expression patterns in hDPSC of the maxillary and mandibular teeth, we demonstrate that *PITX1* has a significantly higher expression in the mandibular posterior teeth compared with the maxillary posterior teeth. The difference in the *PITX1* level is more distinct in the molars than the premolars when comparing the upper and lower teeth. Notably, we propose that the differential gene expression between the maxillary and mandibular teeth should be taken into consideration when investigating the molecular biology of hDPSC in vitro. We also show that RNA-Seq expression analysis of hDPSC is a viable method for identifying genes and pathways underlying the function of dental pulp in permanent teeth. By continuous improvement of our understanding in these areas, we can improve the ways we diagnose and treat pathologies affecting human teeth, whether they arise from genetic or environmental factors, injury, or disease.

## Materials and methods

### Subjects and hDPSC isolation

The research protocol was approved by the Human Ethics Committee (HREC-DCU 2019-062), Faculty of Dentistry, Chulalongkorn University. The experiments were performed in accordance with the Helsinki declaration and relevant guideline and regulations. Informed consent was obtained from all subjects and/or their legal guardians. Ten participants (range 13–31 years old) (4 subjects for RNA-Seq and 6 subjects for qRT-PCR) who did not have any medical problems and had a pair of teeth extracted based on their dental treatment plan were recruited. Five pairs of upper and lower left premolars (teeth 24 and 35) and five pairs of upper and lower left third molars (teeth 28 and 38) from the same side of the same subject were obtained simultaneously. All teeth were healthy without any carious lesions or fillings. Tooth number was designated according to the FDI World Dental Federation notation. The dental pulp tissues were gently removed and explanted. The hDPSC were maintained in Dulbecco’s Modified Eagle Medium (DMEM, Gibco, Waltham, MA, USA) supplemented with 10% fetal bovine serum (FBS) (Gibco), 1% l-glutamine, 100 U/ml penicillin, and 100 μg/ml streptomycin (Gibco). The cells were incubated in a 5% CO_2_ humidified atmosphere at 37 °C^42,43^. The hDPSC from passage 4 were used in the study. The cells were observed under microscope (Zeiss Primovert, Carl Zeiss AG, Oberkochen, Germany) at 10X magnification.

### Flow cytometry

Flow cytometry was used to examine the surface protein expression of mesenchymal stem cell markers^44,45^. The cells were detached using trypsin/EDTA solution to obtain a single cell suspension. The cells were immunostained with primary antibodies conjugated with fluorescent dye, comprising CD44 (Cat No. AM310‐10M, BioGenex, Fremont CA, USA), CD45 (Cat No. AM111‐10M, BioGenex), CD73 (Cat No. 21270733, ImmunoTools, Friesoythe, Germany), CD90 (Cat No. 21270906, ImmunoTools), and CD105 (Cat No. 21271054, ImmunoTools). Flow cytometry analysis was performed using a FACS^Calibur^ Flow cytometer (BD Biosciences, NJ, USA).

### RNA preparation and sequencing

RNA was isolated from the hDPSC at passage 4 on day 7 using an RNeasy Plus Mini kit (Qiagen, Valencia, CA, USA) according to the manufacturer's protocol with DNaseI treatment. The RNA was eluted from the column using nuclease-free water. The RNA quality was examined using a Bioanalyzer (Agilent 2100, Agilent Technologies). Samples with RIN values above 7 across were used. Total RNA (1 μg) was used for mRNA library preparation. The RNA samples from two pairs of opposing premolars (P1, P2) and two pairs of opposing third molars (M1, M2) were sequenced on an Illumina NovaSeq6000 platform (TruSeq stranded mRNA library) with 100 bp paired-end run mode and 40–60 million reads per sample (Macrogen Inc., Seoul, Korea).

### RNA sequencing analysis

The RNA-Seq Alignment program was used to analyze the raw data and the RNA-Seq Differential Expression program (Illumina Inc., San Diego, CA, USA) was used for data analysis after alignment according to the Homo sapiens reference genome (UCSC hg19). The upper teeth were used as a control group and the lower teeth as a comparison group. The mean count was the normalized mean count of the control and comparison groups. Genes with a low mean count of below 10 were excluded. Genes were considered true significance when *p*-value ≤ 0.05 with a false discovery rate (FDR) ≤ 0.05. The genes with true significance and log2 fold change (log2FC) ≤ − 2 or ≥ 2 (exhibiting 4-fold change or more) from each pair of premolar teeth and molar teeth were selected and compared. The results were compared with the RNA-Seq analysis from all 4 pairs. Pathway analysis was performed using Reactome^46^. The RNA sequencing data was deposited in the Gene Expression Omnibus (GEO accession number: GSE193471).

### Quantitative real-time PCR

The RNA-Seq results were validated by qRT-PCR analysis. Total RNA was extracted from hDPSC of the other six pairs of teeth (3 premolars and 3 molars pairs). RNA extraction was performed using an RNeasy Plus Mini Kit (Qiagen) according to the manufacturer’s instructions. The genes that had log2FC ≤ − 2 or ≥ 2 and significant difference between the upper and lower teeth from the RNA-Seq analysis were selected for qRT-PCR. The RNA samples were converted to cDNA using a reverse transcriptase (Promega, Madison, WI, USA). The mRNA level expression was determined using CFX Real-Time PCR Detection Systems (Bio-Rad, Hercules, CA, USA) with primers for *PITX1* (F: GTGGCGTAAGCGCGAGCGTAA, R: GACAGCGGGCTCATGGAGTTGAAG), and *DNAAF4-CCPG1* (F: CCAATTCGACCCTCTGGCAA, R: TGACTGAAACAGTACTCAGCAAAAT). Each experiment was performed in duplicate. The expression levels were normalized to the reference housekeeping gene, *β-actin*, and subsequently calculated using the 2-∆∆Cq method. The bar graphs are presented as mean relative fold change ± standard deviation (SD). Statistical analyses were performed using GraphPad Prism 8.0.2 (GraphPad Software, USA). The Mann Whitney *U* test was used for two-group comparison. The difference between two groups was significant at *p* value of ≤ 0.05.

### Informed consent

Informed consent was obtained from all subjects and/or their legal guardians.

## Supplementary Information

Supplementary Information.

## Data Availability

The datasets generated and/or analyzed during the current study are available in the Gene Expression Omnibus (GEO accession number: GSE193471) and in the supplementary information files.
