# Supplementary material for: Comparative transcriptome profiles of human dental pulp stem cells from maxillary and mandibular teeth
Source: Sci Rep. 2022 May 25;12:8860. doi: 10.1038/s41598-022-12867-1 (PMC9133121; doi:10.1038/s41598-022-12867-1)
Supplement: Supplementary file 1 — Supplementary Information. [file 41598_2022_12867_MOESM1_ESM.pdf]

SUPPLEMENTARY DATA

Comparative transcriptome profiles of human dental pulp stem cells from maxillary and mandibular teeth

Thira Faruangsaeng[1,2], Sermporn Thaweesapphitak[2], Chompak Khamwachirapitak[2], Thantrira Porntaveetus[1,2*], Vorasuk Shotelersuk[3,4]

[1]International Graduate Program in Geriatric Dentistry and Special Patients Care, Faculty of Dentistry, Chulalongkorn University, Bangkok, Thailand

[2]Center of Excellence in Genomics and Precision Dentistry, Department of Physiology, Faculty of Dentistry, Chulalongkorn University, Bangkok 10330, Thailand

[3]Center of Excellence for Medical Genomics, Medical Genomics Cluster, Department of Pediatrics, Faculty of Medicine, Chulalongkorn University, Bangkok, 10330, Thailand

[4]Excellence Center for Genomics and Precision Medicine, King Chulalongkorn Memorial Hospital, the Thai Red Cross Society, Bangkok, 10330, Thailand

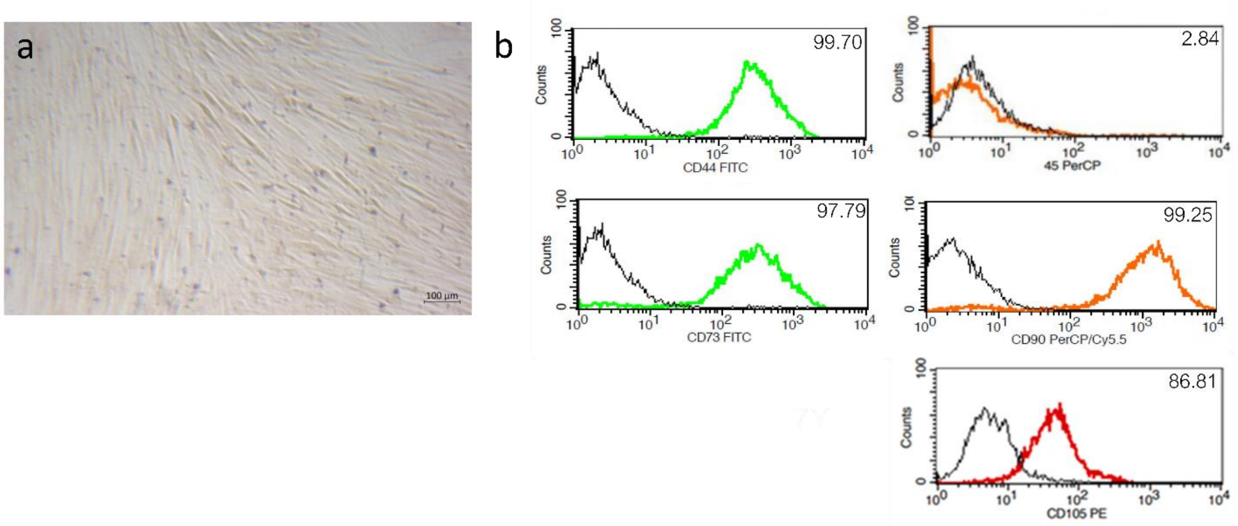

Fig. S1 Human dental pulp stem cell (hDPSC) characterization. a The hDPSC had a spindle shape under microscope at 10X magnification. **b** Flow cytometry showed that hDPSC were positive for CD44 (99.70%), CD73 (97.79%), CD90 (99.25%), and CD105 (86.81%), but negative for CD45 (2.84%).

Table S1 20 top-expression genes in the upper premolars

Genes	Count		
	Upper premolar 1	Upper premolar 2	Mean
1) *FN1*	853954.7	866299.7	863429.6
2) *COL1A2*	536551.2	494620	514913.9
3) *ACTB*	465644.2	424213.7	443986.1
4) *EEF1A1*	350065.3	406808.1	387375.5
5) *TGFBI*	96093.95	550661.8	340760.8
6) *COL1A1*	542881.7	81018.95	294047.1
7) *VIM*	347757.2	197607.3	266072.5
8) *FTH1*	76591.95	389346.4	252911.8
9) *GREM1*	135423.3	255886	208786.4
10) *ACTG1*	232469.7	181235	205113.5
11) *IGFBP5*	123865.5	230236.4	182979.8
12) *COL6A3*	219547.6	150360.6	182289
13) *THBS1*	181930.2	162697.9	172412.9
14) *PENK*	197.1548	289768.2	158039.9
15) *FLNA*	222741	88166.41	151404.8
16) *GAPDH*	134745.4	140879.8	137898.6
17) *PTGS1*	39725.17	201251.9	128332.3
18) *FSTL1*	100987.7	133983	120746.6
19) *FTL*	56742.56	167780.7	114422.1
20) *COL6A2*	84064.44	137829.6	112922.8

Table S2 20 top-expression genes in the lower premolars

Genes	Count		
	Lower premolar 1	Lower premolar 2	Mean
1) *FN1*	731532.75	948013.14	839772.9
2) *COL1A2*	347285.89	720341.07	533813.5
3) *ACTB*	360057.80	348383.84	354220.8
4) *COL1A1*	356972.07	341019.60	348995.8
5) *EEF1A1*	263796.29	366186.36	314991.3
6) *VIM*	289188.06	270188.50	279688.3
7) *TGFBI*	50263.65	411307.27	230785.5
8) *ACTG1*	211085.99	193315.21	202200.6
9) *FTH1*	48299.71	350915.16	199607.4
10) *THBS1*	222719.50	163533.09	193126.3
11) *GREM1*	105511.83	267795.60	186653.7
12) *PENK*	26762.53	329865.62	178314.1
13) *GAPDH*	131272.26	216535.09	173903.7
14) *IGFBP5*	94387.68	250384.91	172386.3
15) *COL6A3*	118588.87	210611.13	164600.0
16) *TIMP3*	45765.42	253683.76	149724.6
17) *FLNA*	178216.23	108365.23	143290.7
18) *FSTL1*	80682.70	140907.00	110795.3
19) *COL6A2*	65960.32	129411.05	97685.7
20) *COL5A2*	58002.94	129550.85	93776.9

Table S3 20 top-expression genes in the upper molars

Genes	Count		
	Upper molar 1	Upper molar 2	Mean
1) *FN1*	989586.8	1011061	1006786
2) *COL1A2*	563030.8	437266.1	510880.9
3) *ACTB*	494335.6	417816.3	462643.4
4) *EEF1A1*	413496.3	317704.8	381988.4
5) *COL1A1*	287593.1	435163	355628.8
6) *VIM*	308996.5	378385.4	341845.8
7) *ACTG1*	258386.7	242629.4	252427.9
8) *GREM1*	280898.1	62583.58	198168.3
9) *COL6A3*	239727	132659.1	193142.9
10) *GAPDH*	203708.8	156111.9	182194.3
11) *FLNA*	129699.5	196587.9	160891.5
12) *TGFBI*	239372.5	56580.89	157917.2
13) *FTH1*	180418.5	110501.2	151807.3
14) *PENK*	220486.8	54093.46	147533.8
15) *COL6A2*	190618.9	95365.19	147446.3
16) *THBS1*	150190.9	124207.4	141744.6
17) *IGFBP5*	164621.3	75364.34	126801.1
18) *TIMP3*	156516.2	58080.13	115865.1
19) *DKK3*	117546.7	97518.97	109565.9
20) *ITGB1*	94351.16	124100.1	108884.1

Table S4 20 top-expression genes in the lower molars

Genes	Count		
	Lower molar 1	Lower molar 2	Mean
1) *FN1*	507727.5	594020	550001.9
2) *COL1A1*	296686.7	571735.2	423519.2
3) *COL1A2*	355850.7	487052.3	416950.9
4) *ACTB*	352479.6	426417	387886.7
5) *EEF1A1*	320701.7	389411.8	355100.3
6) *VIM*	342354.9	224251.5	292256.8
7) *ACTG1*	204374.5	239459.7	221354.8
8) *IGFBP5*	162427.9	245287.6	200383.1
9) *GAPDH*	209489.6	141546	178444.2
10) *COL6A3*	187409.2	133366	164130.6
11) *TGFBI*	211953.2	73935.89	150178.2
12) *FLNA*	128570.8	161127.9	144112.5
13) *THBS1*	77011.13	195886.3	128187
14) *FTH1*	112695.9	138679.7	125137.7
15) *PKM*	119262.2	110590.2	115709.6
16) *DKK3*	84158.7	140901.2	110018
17) *COL6A2*	139416.6	61789.05	103955.8
18) *PENK*	184167.9	224.5692	102578.1
19) *SPARC*	72350.34	137015.6	100980.4
20) *ITGB1*	73315.01	126422.9	96355.57

Table S5 Examples of 20 pathways related to the fifteen commonly expressed genes in the hDPSC of premolars and molars.

Pathways	*p* value	FDR*	Genes related to the pathway
**Extracellular matrix organization**			
- Syndecan interactions	1.43E-09	1.51E-07	*COL1A1*, *COL1A2*, *TGFB1*,*FN1*, *THBS1*
- ECM proteoglycan	3.56E-09	2.49E-07	*COL1A1*, *COL1A2*, *COL6A2*, *COL6A3*, *FN1*, *TGFB1*
- Integrin cell surface interactions	5.87E-09	3.11E-07	*COL1A1*, *COL1A2*, *COL6A2*, *COL6A3 FN1*, *THBS1*
- Non-integrin membrane - ECM interactions	5.67E-08	2.38E-06	*COL1A1*, *COL1A2*, *TGFB1*, *FN1*, *THBS1*
- Extracellular matrix organization	8.48E-07	2.74E-05	*COL1A1*, *COL1A2*, *COL6A2*, *COL6A3*, *FN1*, *TGFB1*, *THBS1*
- Collagen chain trimerization	9.14E-07	2.74E-05	*COL1A1*, *COL1A2*, *COL6A2*, *COL6A3*
- Degradation of extracellular matrix	4.33E-06	9.95E-05	*COL1A1*, *COL1A2*, *COL6A2*, *COL6A3*, *FN1*
- Assembly of collagen fibrils and other multimeric structures	4.79E-06	1.01E-04	*COL1A1*, *COL1A2*, *COL6A2*, *COL6A3*
- Collagen degradation	5.37E-06	1.02E-04	*COL1A1*, *COL1A2*, *COL6A2*, *COL6A3*
- Collagen biosynthesis and modifying enzymes	7.85E-06	1.18E-04	*COL1A1*, *COL1A2*, *COL6A2*, *COL6A3*
- Collagen formation	2.67E-05	3.47E-04	*COL1A1*, *COL1A2*, *COL6A2*, *COL6A3*
**Immune system**			
- Interleukin-4 and interleukin-13 signaling	1.36E-09	1.51E-07	*COL1A2*, *FN1*, *TGFB1*, *VIM*
- Signaling by interleukins	6.51E-06	1.11E-04	*COL1A2*, *FN1*, *TGFB1*, *VIM*
- Cytokine signaling in immune system	4.06E-05	4.87E-04	*COL1A2*, *FN1*, *TGFB1*, *VIM*
**Hemostasis**			
- GP1b-IX-V activation signaling	1.19E-06	3.09E-05	*COL1A1*, *COL1A2*, *FLNA*
- Platelet activation, signaling and aggregation	7.33E-06	1.17E-04	*COL1A1*, *COL1A2*, *FLNA*, *FN1*, *TGFB1*, *THBS1*

**Pathways**	***p* value**	**FDR***	**Genes related to the pathway**
**Signal transduction**			
- MET activates PTK2 Signaling	2.20E-05	3.08E-04	*COL1A1*, *COL1A2*, *FN1*
- Signaling by receptor tyrosine kinases	5.14E-05	5.66E-04	*ACTB, COL1A1*, *COL1A2*, *COL6A2*, *COL6A3*, *FN1*, *THBS1*
- MET promotes cell motility	6.04E-05	6.64E-04	*COL1A1*, *COL1A2*, *FN1*
**Vesicle-mediated transport**			
- Scavenging by class A receptors	7.76E-05	7.76E-04	*COL1A1*, *COL1A2*, *FTH1*

*FDR, false discovery rate.

Table S6 Differential gene expression between upper and lower posterior teeth (2 pairs of premolars and 2 pairs of molars combined). The table showed genes with true significance and log2 fold change ≤−2 or ≥2.

	**Genes**	
	PITX1	***DNAAF4-CCPG1***
Base mean	124.00	42.16
Upper posterior teeth (control)	2.76	0.18
Lower posterior teeth (comparison)	245.23	84.14
Log2 (upper posterior teeth)	1.47	-2.47
Log2 (lower posterior teeth)	7.94	6.39
Log2 fold change	6.47	8.86
p value	9.68E-17*	1.87E-12*
Adjusted p-value (*q*)	1.85E-14**	1.79E-10**

*Significance at $p \leq 0.05$, **Significance at $q \leq 0.05$ (false discovery rate ≤ 0.05)

Table S7 Differential expression analysis between the upper and lower premolars.

	Premolar teeth pairs		
	**Pair P1**	**Pair P2**	**Pairs P1 + P2**
Total gene count	27,914	27,914	27,914
Assessed gene count<br>(excluding genes with low counts)	11,908	15,226	18,772
Gene count with true significance	5,983	4,810	11<br>(Fig. 3a)
Gene count with log2FC ≤−2 or ≥2	62<br>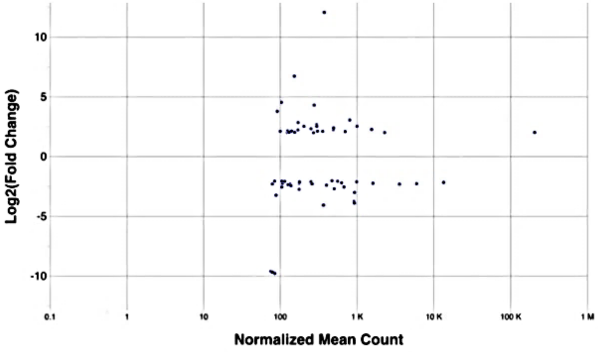	172<br>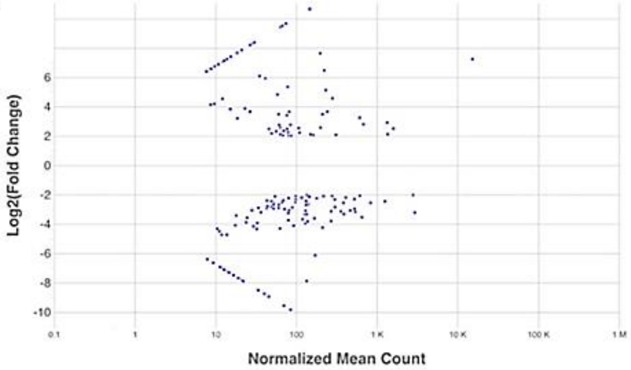	10<br>(Fig. 3b)

Table S8 Differential genes between two pairs of the upper and lower premolars.

The range set was true significance at log2FC ≤−2 or ≥2

No.	Gene	log2 (upper)	log2 (lower)	log2FC	std. err. log2FC	*q* value
1	*SULT1A3*	-2.5	7.05	9.55	1.78	4.07E-04
2	*DNAAF4-CCPG1*	-2.51	6.07	8.58	1.85	8.13E-03
3	*PITX1*	1.52	7.52	6	1.23	3.07E-03
4	*ASB5*	9.05	5.21	-3.84	0.91	4.13E-02
5	*EPHA6*	7.8	3.39	-4.4	0.9	3.07E-03
6	*KIAA0408*	6.32	-2.39	-8.71	1.94	1.46E-02
7	*SPECC1L-ADORA2A*	6.38	-2.39	-8.77	2.08	4.13E-02
8	*PLA2G4B*	6.94	-2.39	-9.32	1.69	2.00E-04
9	*SULT1A4*	6.96	-2.39	-9.35	1.66	2.00E-04
10	*MEF2B*	7.05	-2.39	-9.44	1.7	2.00E-04

Std. err., standard error

Table S9 *PITX1* expression data in the premolars

	Premolar teeth pairs		
	**Pair P1**	**Pair P2**	**Pairs P1 + P2**
Base mean	152.20	23.05	93.35
Upper premolars (control group)	2.84	2.92	2.88
Lower premolars (comparison group)	301.56	43.19	183.82
Log2 (upper premolars)	1.50	1.54	1.52
Log2 (lower premolars)	8.24	5.43	7.52
Log2 (fold change)	6.73	3.89	6.00
p value	3.76E-21*	0.0041*	9.81E-07*
p-adjust value (*q*)	3.20E-20**	0.0154**	3.07E-03**

*Significance at $p \leq 0.05$, **significance at $q \leq 0.05$ (false discovery rate ≤ 0.5), the upper teeth were used as a control group and lower teeth as the comparison group

Table S10 *DNAAF4-CCPG1* expression data in the premolars

	**Premolar teeth pairs**		
	**Pair P1**	**Pair P2**	**Pairs P1 + P2**
Base mean	13.73	63.43	33.64
Upper premolars	0.17	0.18	0.18
Lower premolars	27.29	126.68	67.11
Log2 (upper premolars)	-2.53	-2.48	-2.51
Log2 (lower premolars)	4.77	6.99	6.07
Log2 (fold change)	7.30	9.46	8.58
p value	0.13	4.23E-07*	3.46E-06*
p-adjust value (*q*)	-	3.05E-06**	8.13E-03**

*Significance at $p \leq 0.05$, **significance at $q \leq 0.05$ (false discovery rate ≤ 0.5), the upper teeth were used as a control group and lower teeth as the comparison group

Table S11 Differential expression analysis between the upper and lower molars.

	Molar teeth pairs		
	Pair M1	Pair M2	Pairs M1 + M2
Total gene count	27,914	27,914	27,914
Assessed gene count (excluding genes with low counts)	14,771	11,398	16,412
Gene count with true significance	4,312	2,417	16 (Fig. 4a)
Gene count with log2FC ≤-2 or ≥2	337	45	11 (Fig. 4b)

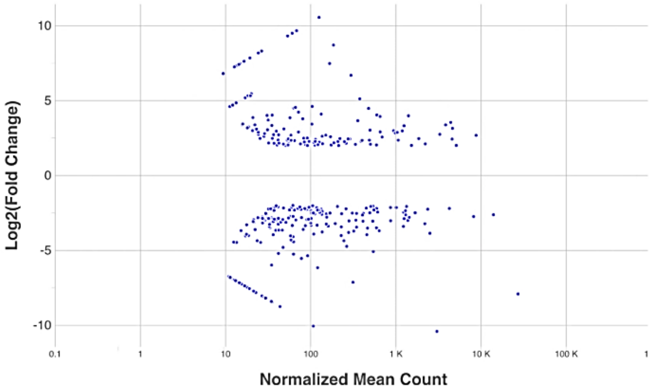

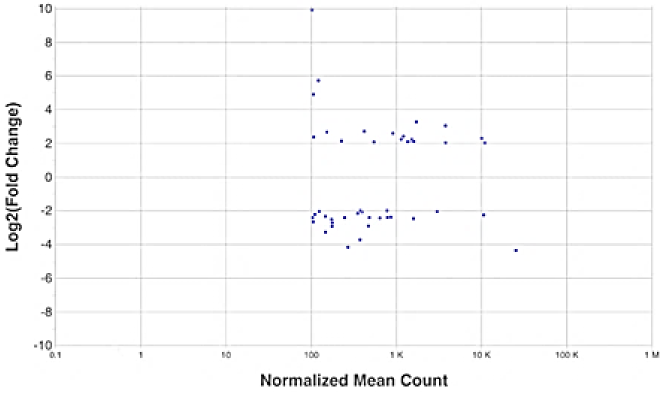

Table S12 Differential genes between two pairs of the upper and lower molars.

The range set was true significance at log2FC ≤−2 or ≥2

No.	Gene	log2(upper)	log2(lower)	log2FC	std. err. log2FC	*q* Value
1	*DNAAF4-CCPG1*	-2.42	6.64	9.06	1.64	1.86E-04
2	*ERV3-1-ZNF117*	-2.43	5.58	8.01	1.83	1.90E-02
3	*C4B_2*	-2.43	5.46	7.89	1.85	2.96E-02
4	*PITX1*	1.36	8.23	6.86	0.87	4.06E-11
5	*NRN1*	3.16	8.18	5.02	1.09	1.04E-02
6	*FOXC2*	7.73	5.25	-2.48	0.6	4.15E-02
7	*NPTX1*	11.28	8.66	-2.62	0.52	1.47E-03
8	*SHOX2*	7.34	4.56	-2.78	0.67	4.15E-02
9	*PIP*	9.16	6.34	-2.82	0.59	5.22E-03
10	*PPARG*	8.98	6.02	-2.97	0.67	1.61E-02
11	*SHISAL1*	8.48	4.29	-4.2	1.02	4.15E-02

Table S13 *PITX1* expression data in the molars

	Molar teeth pairs		
	**Pair M1**	**Pair M2**	**Pairs M1 + M2**
Base mean	184.77	121.07	150.89
Upper molars (control group)	0.88	4.51	2.57
Lower molars (comparison group)	368.66	237.62	299.22
Log2 (upper molars)	-0.18	2.17	1.36
Log2 (lower molars)	8.53	7.89	8.23
Log2 (fold change)	8.71	5.72	6.86
P value	2.17E-14*	2.41E-11*	2.47E-13*
p-adjust value (*q*)	2.81E-13**	3.09E-10**	4.06E-11**

*Significance at $p \leq 0.05$, **Significance at $q \leq 0.05$ (FDR ≤ 0.05)

Table S14 *DNAAF4-CCPG1* expression data in the molars

	Molar teeth pairs		
	**Pair M1**	**Pair M2**	**Pairs M1 + M2**
Base mean	60.62	40.85	49.99
Upper molars (control group)	0.17	0.21	0.19
Lower molars (comparison group)	121.07	81.49	99.79
Log2 (upper molars)	-2.59	-2.26	-2.42
Log2 (lower molars)	6.92	6.35	6.64
Log2 (fold change)	9.50	8.61	9.06
P value	2.14E-08*	0.07	3.39E-08*
p-adjust value (*q*)	1.90E-07**	-	1.86E-04**

Table S15. The rank of *PITX* expression in each tooth samples

Tooth sample	Ranking from total genes (Total gene count: 27,914)	Raking from genes with true significance and log2FC ≤−2 or ≥2
P1 upper	15,529	61/62
P1 lower	9,159	23/62
P2 upper	15,473	141/172
P2 lower	12,523	74/172
M1 upper	16,249	317/337
M1 lower	8,957	48/337
M2 upper	14,846	44/45
M2 lower	9,407	24/45

Table S16 *PITX1* data of premolars from qRT-PCR

	Premolar teeth pairs			Average
	Pair P3	Pair P4	Pair P5	
ΔΔCq (upper)	-0.58	0.75	-0.17	
ΔΔCq (lower)	-5.99	-5.56	-4.77	
ΔCq (upper)	19.23	20.56	19.64	
ΔCq (lower)	13.82	14.25	15.04	
Fold change	63.63	47.07	27.32	46.00

Table S17 *PITX1* data of molars from qRT-PCR

	Molar teeth pairs			Average
	Pair M3	Pair M4	Pair M5	
ΔΔCq (upper)	-0.99	1.90	-0.91	
ΔΔCq (lower)	-7.58	-4.86	-5.82	
ΔCq (upper)	18.17	21.06	18.25	
ΔCq (lower)	11.59	14.31	13.35	
Fold change	190.68	28.94	56.30	91.97
